# Peptide inhibition of human cytomegalovirus infection

**DOI:** 10.1186/1743-422X-8-76

**Published:** 2011-02-22

**Authors:** Lilia I Melnik, Robert F Garry, Cindy A Morris

**Affiliations:** 1 Graduate Program in Biomedical Sciences and Department of Microbiology and Immunology, Tulane University, 1430 Tulane Avenue, New Orleans, LA, 70112 USA

## Abstract

**Background:**

Human cytomegalovirus (HCMV) is the most prevalent congenital viral infection in the United States and Europe causing significant morbidity and mortality to both mother and child. HCMV is also an opportunistic pathogen in immunocompromised individuals, including human immunodeficiency virus (HIV)- infected patients with AIDS, and solid organ and allogeneic stem cell transplantation recipients. Current treatments for HCMV-associated diseases are insufficient due to the emergence of drug-induced resistance and cytotoxicity, necessitating novel approaches to limit HCMV infection. The aim of this study was to develop therapeutic peptides targeting glycoprotein B (gB), a major glycoprotein of HCMV that is highly conserved across the *Herpesviridae *family, that specifically inhibit fusion of the viral envelope with the host cell membrane preventing HCMV entry and infection.

**Results:**

Using the Wimley-White Interfacial Hydrophobicity Scale (WWIHS), several regions within gB were identified that display a high potential to interact with lipid bilayers of cell membranes and hydrophobic surfaces within proteins. The ability of synthetic peptides analogous to WWIHS-positive sequences of HCMV gB to inhibit viral infectivity was evaluated. Human foreskin fibroblasts (HFF) were infected with the Towne-GFP strain of HCMV (0.5 MOI), preincubated with peptides at a range of concentrations (78 nm to 100 μM), and GFP-positive cells were visualized 48 hours post-infection by fluorescence microscopy and analyzed quantitatively by flow cytometry. Peptides that inhibited HCMV infection demonstrated different inhibitory concentration curves indicating that each peptide possesses distinct biophysical properties. Peptide 174-200 showed 80% inhibition of viral infection at a concentration of 100 μM, and 51% and 62% inhibition at concentrations of 5 μM and 2.5 μM, respectively. Peptide 233-263 inhibited infection by 97% and 92% at concentrations of 100 μM and 50 μM, respectively, and 60% at a concentration of 2.5 μM. While peptides 264-291 and 297-315, individually failed to inhibit viral infection, when combined, they showed 67% inhibition of HCMV infection at a concentration of 0.125 μM each.

**Conclusions:**

Peptides designed to target putative fusogenic domains of gB provide a basis for the development of novel therapeutics that prevent HCMV infection.

## Introduction

Human cytomegalovirus (HCMV) is a ubiquitous opportunistic pathogen that belongs to the *Betaherpesviridae*. The virulence of this pathogen is directly linked to the immune status of its host. Primary HCMV infection is generally asymptomatic in immunocompetent individuals, although it causes a mononucleosis-like syndrome in some. After primary HCMV infection, the virus establishes lifelong latency and periodically reactivates with notable pathological consequences. In contrast, HCMV infection in immunocompromised patients such as AIDS patients and solid organ and allogeneic stem cell transplantation recipients causes serious disease [1]. Primary infection of women during or right before pregnancy with HCMV is the most common cause of congenital viral infection leading to significant morbidity and mortality. Congenital HCMV infection is also associated with spontaneous abortion, premature delivery, intrauterine growth restriction (IUGR), and pre-eclampsia. The risk of primary infection in a seronegative mother is 1 to 4%, which carries a 30 to 40% risk of congenital infection [2,3]. The majority of congenitally infected babies are asymptomatic at birth; however, 10 to 17% subsequently develop hearing defects or neurodevelopmental sequelae [4]. Although the most serious clinical sequelae are seen in cases where a mother acquires a primary infection during pregnancy, downstream side effects are also seen in cases where latent HCMV is reactivated [5] and where a mother is reinfected with a different strain of the virus [6].

HCMV has a double-stranded DNA genome of 235 kb encoding approximately 165 genes [7]. It has a very broad cellular tropism resulting in potential infection of nearly every organ system. The ability of HCMV to enter a wide range of cell types involves a complex interaction between several viral envelope glycoproteins and host cell surface receptors, although the entry of herpesviruses into host cells is still poorly understood. The HCMV virion envelope contains at least 20 virus-encoded glycoproteins that are involved in cell attachment and penetration [8]. Of these, glycoprotein B (gB) is the most abundant glycoprotein [9] and is highly conserved among the *Herpesviridae *[10]. Glycoprotein B plays a critical role in the HCMV entry process. Initially, gB along with gM/gN, is involved in tethering of virions to heparan sulfate proteoglycans (HSPG) on the surface of host cells. The short interaction of HCMV with HSPG is followed by more stable interactions with one or more viral cellular receptors, namely epidermal growth factor receptor (EGFR) [11], platelet-derived growth factor receptor (PDGFR) [12], and toll-like receptor TLR-2 [13]. Glycoprotein B also interacts with integrin αvβ3, a coreceptor that enhances HCMV entry [14]. Integrins are known to synergise with EGFR as well as with other receptors to activate signal transduction pathways [15-17]. To complete the entry process, both viral and cellular membranes fuse, allowing the release of virion-associated tegument and capsid proteins into the cytoplasm. This final step of viral entry into host cells requires gB and the gH/gL complex [18-21].

Antibodies to HCMV gB have been shown not only to block penetration of virions into cells, but also to limit cell-to-cell infection, implying that gB plays a role in virion penetration into cells, cell-to-cell transmission, as well as fusion of infected cells [20,22]. Recently, Isaacson and coworkers used genetic complementation to confirm that gB is required for the fusion of viral and cellular membranes, virus entry, and cell-to-cell spread of HCMV [23]. The importance of gB for viral infection suggests that this viral envelope protein may be a rational target for novel drug design.

HCMV infection is highly prevalent in the population due to the ability of the virus to efficiently transmit between hosts that harbour and periodically shed the virus. HCMV is transmitted through direct exposure to infected bodily secretions, including saliva, urine and breast milk. Following infection, HCMV enters the bloodstream and spreads to various organs including kidney, liver, spleen, heart, brain, retina, esophagus, inner ear, lungs, colon, and salivary glands [24]. The ability of HCMV to infect a wide variety of cell types is not due to the presence of high plasma levels of extracellular virus, but is primarily due to cell-to-cell transmission between mononuclear phagocytes (possibly macrophages or dendritic cell precursors) and uninfected tissues [25].

The lack of a successful HCMV vaccine as well as the toxicity and drug-induced resistance associated with current therapeutics for HCMV indicate that this virus continues to pose a significant public health problem. Current treatments for HCMV disease target viral replication and can fail due to the emergence of drug-resistant virus variants and induction of adverse effects. Hence, a new approach in drug design against HCMV is required [26,27]. Since HCMV and other herpesviruses establish a lifelong latency in humans, antiviral therapy that inhibits viral entry may serve as an alternative to the already existing and inadequate therapeutic agents. Here, we report the design, development and characterization of peptides that specifically inhibit viral infection and/or entry as a novel approach to prevent HCMV infection.

## Results

### HCMV gB is a likely class III viral fusion protein

Structural studies place herpes simplex virus type 1 gB-1 [28] and Epstein-Barr virus gB into class III viral fusion proteins (VFP) [29], which also includes VSV G [30], members of the GP64 superfamily (baculovirus and thogotovirus) [31] and tentatively bornavirus G [32]. Because gB is the most highly conserved envelope protein amongst the mammalian and avian herpesvirues [25,33], gB of HCMV is likely to be a class III VFP and shares structural features with gB of other members of the *Herpesviridae*. Class III viral fusion proteins share certain characteristics found in class I or class II viral fusion proteins. The class III viral fusion proteins contain an extended α-helix that trimerizes in the post-fusion forms of the proteins [28,30,34], as has been well-documented for the post-fusion forms of the class I viral fusion proteins of orthomyxoviruses, retroviruses, paramyxoviruses, arenaviruses, and coronaviruses [32]. Similarly, the class II viral fusion proteins of flaviviruses and alphaviruses contain a fusion domain comprised principally of β-sheets and "fusion loops." Class III viral fusion proteins also possess a fusion domain, as well as several other features of class II viral fusion proteins, suggesting that these two classes of proteins may share a common progenitor [31].

**Figure 1:**
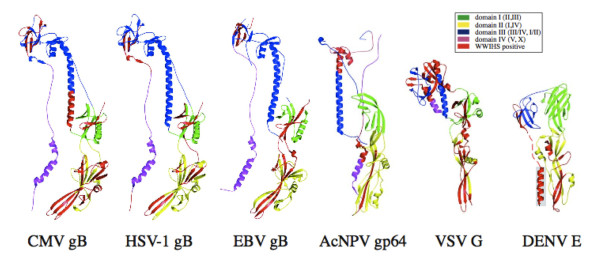
**Wimley-White interfacial hydrophobicity scale score-positive sequences in class II and III viral fusion proteins**. Sequences of a representative class II viral fusion protein (Dengue virus E) and of class III viral fusion proteins with high potential to interface with lipid membranes (red) were identified using Membrane Protein Explorer software (MpeX version 3.0). As discussed in the text, a class III domain nomenclature is used here that can apply to both class II and III viral fusions proteins. The alternative domain number schemes used by Roche *et al*. and Heldwein *et al*. are noted in parentheses. The Dengue virus (DENV E) stem domain sequence that was not included in the protein used to determine the crystal structure has been added. The DENV stem has a positive WWIHS scale score, and corresponds to a previously determined inhibitor of DENV and West Nile virus [35].

The class III domain nomenclature used here can apply to both class II and class III viral fusion proteins: domain I (green), domain II (yellow), domain III (blue), domain IV (stem domain, indigo) (Figure 1). This unified nomenclature assigns domain II appellation to the following: VSV G domain IV in the nomenclature of Roche *et al*. [30], HSV-1 gB-1 and baculovirus gp64 domain I in the nomenclature of Heldwein *et al*. [28] and Kadlec *et al*. [31] as the class III fusion domain, which is structurally similar to class II viral fusion proteins. In addition to minor adjustments in the ends of domains, the current class III viral fusion protein numbering also combines two interacting domains into domain III (I + II in Roche's VSV G nomenclature, III + IV in Heldwein's HSV-1 gB-1 nomenclature and Kadlec's baculovirus nomenclature).

### Identification of HCMV gB inhibitory peptides

The Wimley-White Interfacial Hydrophobicity Scale (WWIHS) is an experimentally determined hydrophobicity scale that provides a quantitative description of a protein partitioning and folding into membrane interfaces. WWIHS score-positive sequences may also interact with hydrophobic surfaces within proteins, and are often sequestered within pre-fusion forms of viral fusion proteins. In addition to similarities in the overall structure of the post-fusion forms of class III VFP, there are additional similarities in the distribution of WWIHS-positive sequences (Figure 1, red). The similarities include at least one extended "fusion loop" in the fusion domain (domain II), and one or more WWIHS score-positive sequences in domain III. With the exception of the ACNPV GP64, each of these proteins contains another WWIHS positive domain II sequence near the "hinge" region adjacent to the domain. Herpesvirus gB proteins have an additional WWIHS scale score-positive sequence in domain I. In the case of class II and III viral fusion proteins, the fusion loops in the fusion domain often contain sequences with positive WWIHS scores.

**Figure 2:**
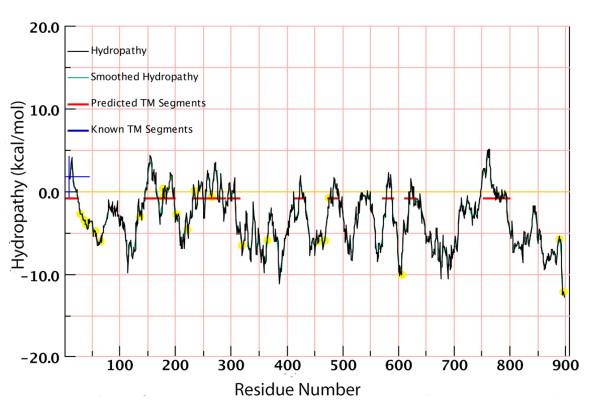
**Determination of regions within gB that display a high propensity to interact with the lipid surface of cell membranes by using Wimley-White Interfacial Hydrophobicity Scale (WWIHS)**. WWIHS identifies segments of proteins that prefer a transbilayer helix conformation to an unfolded interfacial location. We used the Interface Scale of the Membrane Protein explorer (MpeX version 3.0) computer program to identify these particular segments of HCMV gB. The Interface scale measures a residue's free energy of transfer within an unfolded polypeptide chain from water to a phosphocholine bilayer. We identified nine segments of HCMV gB that display high propensity to interact with the lipid surface of cell membrane, and designed peptides, ranging from 19 to 31 amino acids in length, that are analogous to the identified regions of gB.

Previous studies have suggested that synthetic peptides corresponding to or overlapping with sequences in viral fusion proteins that have positive WWIHS scores can sometimes serve as viral entry inhibitors [35-50]. For example, Enfurvitide (Fuzeon) is a 36-amino acid peptide that overlaps with a WWIHS score-positive sequence in the transmembrane protein (TM) of HIV-1, and prevents viral fusion and entry of the virus. To identify regions of HCMV gB that have a high propensity to interact with the lipid bilayer of cell membranes and which potentially may serve as HCMV entry inhibitors, we employed Membrane Protein eXplorer version 3.0 (http://blanco.biomol.uci.edu/mpex), a computer program based on the WWIHS. Nine sequences with significant positive WWIHS scores were identified (Figure 2). As expected, several of these WWIHS sequences corresponded to the predicted fusion domain of HCMV gB, including the predicted fusion loops. One of the WWIHS score-positive sequences spanned amino acids 146 to 200 (peptide 146-200) that had a ΔG score of 4.33 kcal/mol. This sequence was split into two smaller peptides: 146-173 and 174-200. A second large segment within the fusion domain of HCMB gB had a ΔG score of 3.39, and was split, consequently, into smaller peptides: 233-263 and 264-291. An additional peptide, 297-315 corresponding to another fusion domain sequence, was also synthesized, along with an additional 4 peptides corresponding to other WWIHS-positive domains of HCMV gB. To prevent dimer formation, cysteines were replaced with alanines.

Nine synthetic peptides corresponding to sequences with significant WWIHS scores were synthesized and examined for their ability to inhibit HCMV infection of HFF cells. Peptides that were most effective are presented here (Table 1). All synthetic peptides were tested at the following concentrations: 100 μM, 50 μM, 25 μM, 10 μM, 5 μM, 2.5 μM, 1.25 μM, 0.625 μM, 0.3125 μM, 0.156 μM, and 0.078 μM. HFF were seeded at a density of 3.5×10^5 ^cells in each well of a 24-well plate 24 hours prior to infection. HFF were washed with 1× DPBS and mock- or virus-infected for 90 minutes at RT with the Towne-GFP strain of HCMV (0.5 MOI) preincubated with different concentrations of inhibitory peptides at 37°C for 90 minutes. After infection, virus was removed and Dulbecco's modified Eagle medium (DMEM) supplemented with 10% fetal bovine serum (FBS), penicillin G (100 U/mL), streptomycin (100 mg/mL), and GlutaMAX (2 mM) was added to each well and cells were incubated at 37°C for 48 hours. GFP-positive cells were visualized 48 hours post-infection by fluorescence microscopy and then quantified using flow cytometry.

**Table 1:** Amino acid sequences of HCMV gB peptides

Peptide	Amino acid sequence	Position
174-200	WEIHHINKFAQAYSSYSRVIGGTVFVA	174-200
233-263	WHSRGSTWLYRETANLNAMLTITTARSKYPY	233-263
264-291	HFFATSTGDVVYISPFYNGTNRNASYFG	264-291
297-315	FFIFPNYTIVSDFGRPNAA	297-315

HCMV gB peptides were synthesized based on the amino acid sequence determined from GenBank accession no. DAA00160 (Human Herpesvirus 5 strain AD169).

**Figure 3:**
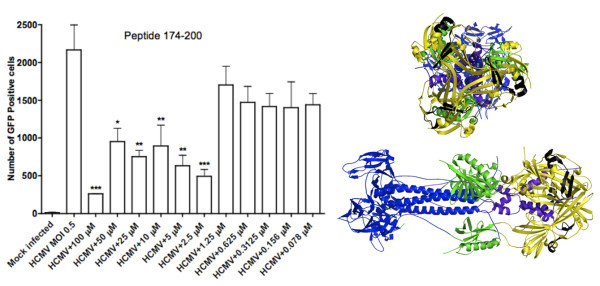
**Inhibition of HCMV infection by peptide 174-200**. HFF were seeded at a density of 3.5×10^5 ^cells in each well of a 24-well plate 24 hours prior to infection. The inhibitory effect of peptide 174-200 was evaluated by infecting human foreskin fibroblasts (HFF) with the Towne-GFP strain of HCMV (0.5 MOI) preincubated with different concentrations of inhibitory peptide 174-200 at 37°C for 90 minutes. GFP-positive cells were visualized 48 hours post-infection by fluorescence microscopy and then quantified using flow cytometry. Significant reductions in the number of GFP-positive cells compared to HCMV infected cells are denoted by a * (p < 0.05), ** (p < 0.01), and *** (p < 0.001, determined using one-way ANOVA and Tukey's post test). All structural figures of HSV-1 gB in the post-fusion configuration were generated using MacPyMOL [56] and FreeHand (Macromedia). Different domains of gB are shown in the ribbon structures: yellow-fusion domain II, purple-stem, green-domain I, and blue-domain III with extended α-helices, which are involved in trimerization. Peptides targeting different domains of HSV-1 gB that correspond to HCMV gB domains are shown in black.

**Figure 4:**
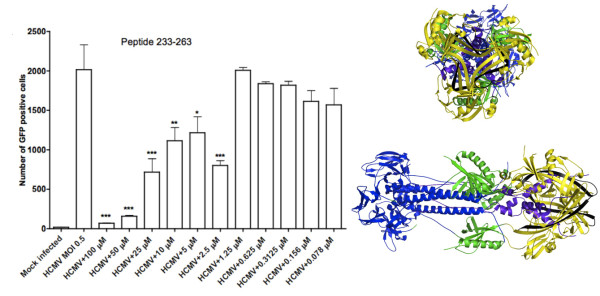
**Inhibition of HCMV infection by peptide 233-263**. HFF were seeded at a density of 3.5×10^5 ^cells in each well of a 24-well plate 24 hours prior to infection. The inhibitory effect of peptide 233-263 was evaluated by infecting human foreskin fibroblasts (HFF) with the Towne-GFP strain of HCMV (0.5 MOI) preincubated with different concentrations of inhibitory peptide 233-263 at 37°C for 90 minutes. GFP-positive cells were visualized 48 hours post-infection by fluorescence microscopy and then quantified using flow cytometry. Significant reductions in the number of GFP-positive cells compared to HCMV infected cells are denoted by a * (p < 0.05), ** (p < 0.01), and *** (p < 0.001, determined using one-way ANOVA and Tukey's post test). All structural figures of HSV-1 gB in the post-fusion configuration were generated using MacPyMOL [56] and FreeHand (Macromedia). Different domains of gB are shown in the ribbon structures: yellow-fusion domain II, purple-stem, green-domain I, and blue-domain III with extended α-helices, which are involved in trimerization. Peptides targeting different domains of HSV-1 gB that correspond to HCMV gB domains are shown in black.

**Figure 5:**
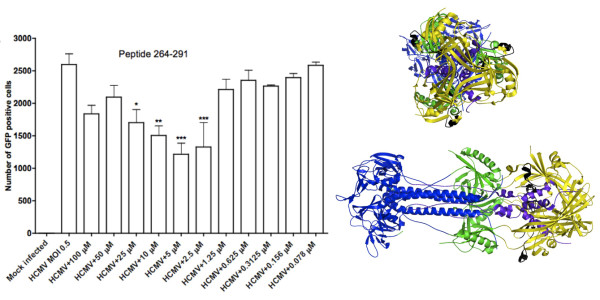
**Inhibition of HCMV infection by peptide 264-291**. HFF were seeded at a density of 3.5×10^5 ^cells in each well of a 24-well plate 24 hours prior to infection. The inhibitory effect of peptide 264-291 was evaluated by infecting human foreskin fibroblasts (HFF) with the Towne-GFP strain of HCMV (0.5 MOI) preincubated with different concentrations of inhibitory peptide 264-291 at 37°C for 90 minutes. GFP-positive cells were visualized 48 hours post-infection by fluorescence microscopy and then quantified using flow cytometry. Significant reductions in the number of GFP-positive cells compared to HCMV infected cells are denoted by a * (p < 0.05), ** (p < 0.01), and *** (p < 0.001, determined using one-way ANOVA and Tukey's post test). All structural figures of HSV-1 gB in the post-fusion configuration were generated using MacPyMOL [56] and FreeHand (Macromedia). Different domains of gB are shown in the ribbon structures: yellow-fusion domain II, purple-stem, green-domain I, and blue-domain III with extended α-helices, which are involved in trimerization. Peptides targeting different domains of HSV-1 gB that correspond to HCMV gB domains are shown in black.

**Figure 6:**
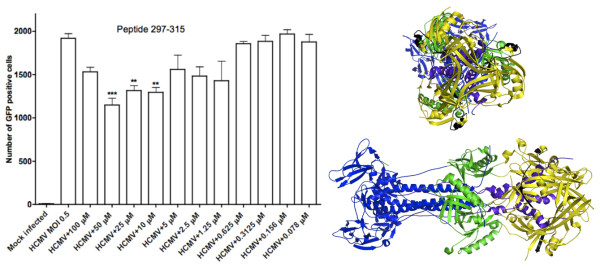
**Inhibition of HCMV infection by peptide 297-315**. HFF were seeded at a density of 3.5×10^5 ^cells in each well of a 24-well plate 24 hours prior to infection. The inhibitory effect of peptide 297-315 was evaluated by infecting human foreskin fibroblasts (HFF) with the Towne-GFP strain of HCMV (0.5 MOI) preincubated with different concentrations of inhibitory peptide 297-315 at 37°C for 90 minutes. GFP-positive cells were visualized 48 hours post-infection by fluorescence microscopy and then quantified using flow cytometry. Significant reductions in the number of GFP-positive cells compared to HCMV infected cells are denoted by a * (p < 0.05), ** (p < 0.01), and *** (p < 0.001, determined using one-way ANOVA and Tukey's post test). All structural figures of HSV-1 gB in the post-fusion configuration were generated using MacPyMOL [56] and FreeHand (Macromedia). Different domains of gB are shown in the ribbon structures: yellow-fusion domain II, purple-stem, green-domain I, and blue-domain III with extended α-helices, which are involved in trimerization. Peptides targeting different domains of HSV-1 gB that correspond to HCMV gB domains are shown in black.

**Figure 7:**
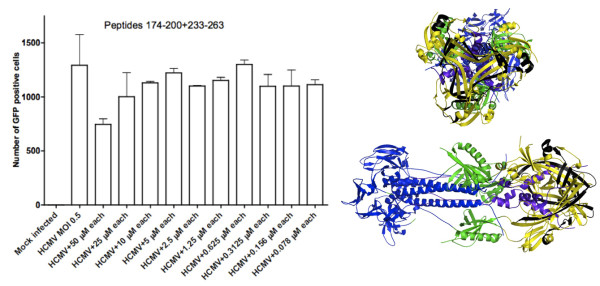
**Additive effect of HCMV gB peptides 174-200 and 233-263 on HCMV infection**. HFF were seeded at a density of 3.5×10^5 ^cells in each well of a 24-well plate 24 hours prior to infection. The ability of peptides 174-200 and 233-263 to work together and their effect on inhibition of virus infection was evaluated by infecting human foreskin fibroblasts (HFF) with the Towne-GFP strain of HCMV (0.5 MOI) preincubated with different concentrations of inhibitory peptides at 37°C for 90 minutes. GFP-positive cells were visualized 48 hours post-infection by fluorescence microscopy and then quantified using flow cytometry. Significant reductions in the number of GFP-positive cells compared to HCMV infected cells are denoted by a * (p < 0.05), ** (p < 0.01), and *** (p < 0.001, determined using one-way ANOVA and Tukey's post test). All structural figures of HSV-1 gB in the post-fusion configuration were generated using MacPyMOL [56] and FreeHand (Macromedia). Different domains of gB are shown in the ribbon structures: yellow-fusion domain II, purple-stem, green-domain I, and blue-domain III with extended α-helices, which are involved in trimerization. Peptides targeting different domains of HSV-1 gB that correspond to HCMV gB domains are shown in black.

**Figure 8:**
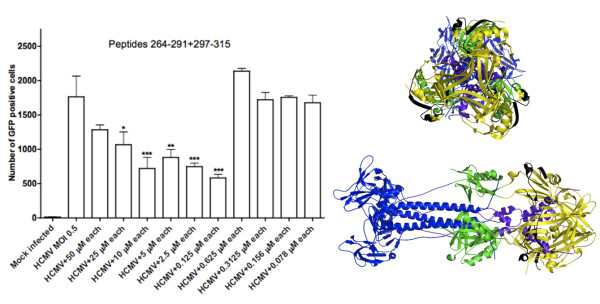
**Additive effect of HCMV gB peptides 264-291 and 297-315 on HCMV infection**. HFF were seeded at a density of 3.5×10^5 ^cells in each well of a 24-well plate 24 hours prior to infection. The ability of peptides 264-291 and 297-315 to work together and their effect on inhibition of virus infection was evaluated by infecting human foreskin fibroblasts (HFF) with the Towne-GFP strain of HCMV (0.5 MOI) preincubated with different concentrations of inhibitory peptides at 37°C for 90 minutes. GFP-positive cells were visualized 48 hours post-infection by fluorescence microscopy and then quantified using flow cytometry. Significant reductions in the number of GFP-positive cells compared to HCMV infected cells are denoted by a * (p < 0.05), ** (p < 0.01), and *** (p < 0.001, determined using one-way ANOVA and Tukey's post test). All structural figures of HSV-1 gB in the post-fusion configuration were generated using MacPyMOL [56] and FreeHand (Macromedia). Different domains of gB are shown in the ribbon structures: yellow-fusion domain II, purple-stem, green-domain I, and blue-domain III with extended α-helices, which are involved in trimerization. Peptides targeting different domains of HSV-1 gB that correspond to HCMV gB domains are shown in black.

**Figure 9:**
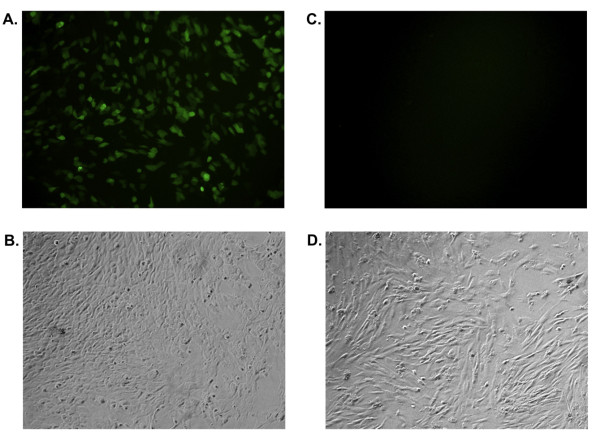
**Bright light and fluorescent images representing the ability of peptide 233-263 to inhibit infection by the Towne-GFP strain of HCMV**. (A-B) Representative fluorescent and bright light microscopic images of HFF infected with the Towne-GFP strain of HCMV (0.5 MOI). (C-D). Representative fluorescent and bright light images of HFF infected with the Towne-GFP strain of HCMV (0.5 MOI) preincubated with peptide 233-263 at a concentration of 100 μM.

Peptide 174-200, for instance, demonstrated 80% inhibition of viral infection at a concentration of 100 μM, and 51% and 62% inhibition at concentrations of 5 μM and 2.5 μM, respectively (Figure 3). Peptide 233-263 inhibited viral infection by 97% and 92% at concentrations 100 μM and 50 μM, respectively, and by 60% at a concentration of 2.5 μM (Figure 4). The scrambled peptide (control), of peptide 233-263, was unable to inhibit HCMV infection significantly (data not shown). While peptide 264-291, alone, showed inhibition of 70.5%, at a concentration of 5 μM (Figure 5), peptide 297-315 tested alone showed 40% inhibition at a concentration of 50 μM (Figure 6). None of the remaining peptides showed significant inhibition of HCMV infection at any of the concentrations tested (data not shown). In addition to testing individual peptides in viral infectivity assays, peptides were similarly tested in combination. Interestingly, when peptides 174-200 and 233-263 were tested together, no significant inhibitory effect was shown (Figure 7). On the contrary, peptides 264-291 and 297-315 tested together displayed 67% inhibition at a concentration of 0.125 μM each (Figure 8). Representative fluorescent and bright light images of HFF cells infected with the Towne-GFP strain of HCMV (0.5 MOI) preincubated with or without peptide 233-263 at a concentration of 100 μM were taken 48 hours post-infection (Figure 9).

## Discussion

The WWIHS is a computational approach, based upon an experimentally determined algorithm to estimate the propensity of an amino acid sequence to interact with lipid membrane interfaces [49]. Using this method, we identified several regions of HCMV gB with high interfacial hydrophobicity. Peptides that are analogous to several of these regions inhibited HCMV infectivity at low μM concentrations (Figure 3, 4, 5, 6, 7 and 8). When tested in combination certain combinations of peptides (peptides 264-291 and 297-315) displayed increased inhibition of infectivity at concentrations of 125 nM (Figure 8). These results suggest that the HCMV inhibitory peptides identified here may serve as the basis for potential antiviral therapies. The success of the inhibition of fusion greatly depends, not only on biophysical properties of synthesized peptides and their concentrations, but also on the size and shape of the binding pocket of HCMV gB. It is possible that the potency of the peptides, either alone or in combination, can be increased by modifying the sequence of the peptides or by conjugating the peptide(s) to other molecules.

Akkarawongsa and coworkers prepared a library of overlapping peptides homologous to the ectodomain of Herpes Simplex Virus Type 1 (HSV-1) gB-1 and screened for the ability of these peptides to block infection [50]. Seven out of 138 15-mer peptides inhibited infection by more than 50% at a concentration of 100 μM. Three peptides (gB131, gB122 and gB94) with 50% effective concentrations below 20 μM were studied further. Peptide gB131 (residues 681 to 695 in HSV-1 gB-1) was identified as a specific entry inhibitor (EC_50_, ~12 μM). The gB122 peptide (residues 636 to 650 in gB-1) blocked viral entry (EC_50_, ~18 μM), protected cells from infection (EC_50_, ~72 μM), and inactivated virions in solution (EC_50_, ~138 μM). Of the seven inhibitory peptides identified in the Akkarawongsa *et al*. study, three of them, corresponding to residues 346 to 360 (gB64), 436 to 450 (gB82), and 636 to 650 (gB122), were in regions of HSV-1 gB-1 with positive WWIHS scores. The success of their study affirms our strategy of targeting the WWIHS score-positive sequences as inhibitors of HCMV. Two overlapping peptides spanning the residues 496 to 510 (gB94) and 501 to 515 (gB95) were not in WWIHS score-positive sequence, but the analogous sequence in HCMV is WWIHS score-positive. Additionally, comparisons across viral families could potentiate identification of entry inhibitors. For instance, HSV-1 inhibitory peptide gB131 corresponding to domain IV, also known as a stem, is analogous to the Hrobowski dengue inhibitory peptide for the class II viral fusion proteins [35] (Figure 1).

**Figure 10:**
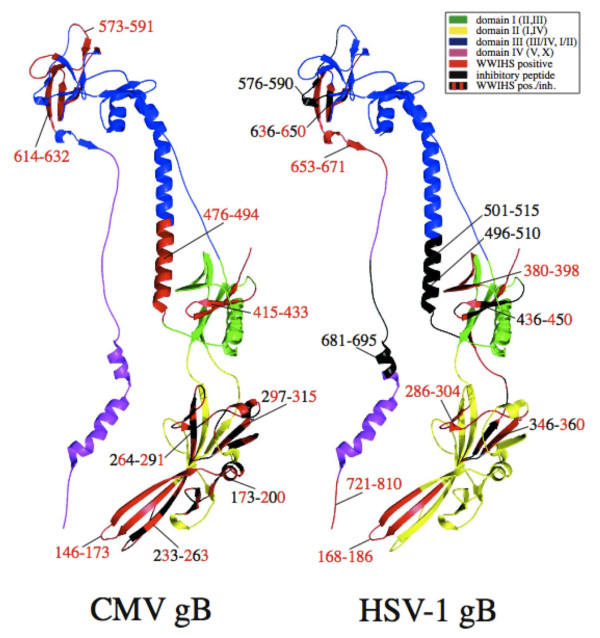
**Comparison of HSV-1 gB-1 inhibitory peptide and HCMV gB inhibitory peptides**. Of the seven HSV-1 gB-1 inhibitory peptides (346-360, 436-450, 496-510, 501-515, 576-590, 636-650, 681-6950) [50', three peptides (346-360, 436-450, 636-650) are both inhibitory and WWIHS score-positive. Two overlapping inhibitory peptides (496-510 and 501-515) are not in WWIHS score-positive sequences, but the analogous sequence in HCMV is WWIHS score-positive. The most potent inhibitors of HCMV infection are all in domain II (174-200, 233-263, 264-291, 297-315). HSV-1 inhibitory peptide gB64 (346-360) corresponds to this region of HCMV gB.

The most potential inhibitors of HCMV infection were all in domain II. HSV-1 inhibitory peptide gB64 (residues 346 to 360) identified by Akkarawongsa *et al*. corresponds to this region, with HCMV inhibitory peptide 297-315 being the analogous peptide (Figure 10). It is possible that some of the inhibitory peptides identified for HSV-1 could be optimized to work for HCMV and vice versa. The fusion domain of HCMV gB may be the initial site of interaction directly with the lipids of the cell bilayer [31]. The HCMV inhibitory peptides could competitively block these interactions, or trigger downstream fusion events, and conformational changes in gB, prematurely. Inhibitory peptides corresponding to other domains likely do not block lipid interactions. Inhibitory peptides may employ several mechanisms of action, and therefore those that are analogous to domain III could block receptor interactions.

As the result of this study we found that the effects of several of the HCMV inhibitory peptides did not follow a linear dose-dependent inhibition curve. This trend was also reported previously describing peptide inhibitors of Dengue and West Nile viruses [35]. Possibly, when the concentration of one or more peptides is too high, the peptides may self-associate, preventing them from interfering with virus infectivity.

In addition to screening synthetic peptides for their ability to inhibit HCMV infection alone, we tested some peptides in combination. It is possible that peptides 264-291 and 297-315 block HCMV entry at distinct steps in the fusion process. Any two peptides that work additively to block the fusion of the virion with the host cell membrane must have unique amino acid sequences, biophysical properties and be present at certain concentrations that will allow them to interact with each other, with gB and, possibly, with other glycoproteins that are instrumental in the fusion event. Peptides 174-200 and 233-263 tested together showed only 42% inhibition at the concentration of 50 μM each (Figure 7), and did not work in an additive fashion. This result may be due to peptide-peptide interactions that do not allow interference with gB trimer formation and with necessary conformational changes of the virion required for successful fusion to occur.

Several drugs, including ganciclovir, its oral prodrug valganciclovir, foscarnet, cidofovir, and fomivirsen have been approved for the treatment of HCMV-associated disease. All of these drugs, with the exception of fomivirsen, have a common target, the viral DNA polymerase [26]. The above listed anti-HCMV drugs provoke not only drug-specific side effects, which include leukopenia, thrombocytopenia, anemia, bone marrow hypoplasia, diarrhea, and renal toxicity, but also the emergence of clinically relevant drug-resistant HCMV [27]. New drugs that are more efficacious and are not toxic in treatment of HCMV infection are urgently needed.

The inhibitory peptides identified here can serve as the basis for the development of a novel therapeutic against HCMV. These antiviral agents could be used as an antiviral treatment to reduce the viral load in pregnant women and neonates. It is not clear how HCMV infects the fetus during pregnancy, but some studies demonstrate that placental infection with HCMV occurs before the transmission of the virus to the fetus and suggest that the placenta plays a role in vertical transmission of HCMV from mother to fetus. Also, placental viral infection has been implicated in spontaneous abortion during early pregnancy that occurs in fifteen percent of women with primary HCMV infection. Placental pathology as a result of HCMV infection during pregnancy may also cause premature delivery, intrauterine growth restriction (IUGR), or pre-eclampsia [51-54].

Enfurvitide, a 36-amino acid peptide also known as Fuzeon works by inhibiting the structural rearrangement of HIV-1 gp41 to block the fusion of HIV-1 virions with their target cell membrane. Brennan-Benson *et al*. showed that Fuzeon prevents vertical transmission of HIV-1 in pregnancy, but does not cross the placenta [55]. We have shown that some peptides are effective at preventing HCMV infection. Those lead peptides will be studied further and modified to increase their efficacy, solubility and delivery.

Currently available drugs to treat HCMV infection are not approved to treat pregnant women due to their potential high toxicity. HCMV infected neonates are treated with these toxic compounds only in cases of high morbidity. The antiviral peptide-based agents that may be developed as a result of this study would not require activation by virally encoded proteins, further phosphorylation by cellular enzymes or incorporation into the growing viral DNA by viral DNA polymerase as the current therapeutics do. Such peptide therapeutics would be predicted not to provoke drug-induced resistance that is a significant problem with existing FDA-approved therapeutics to treat HCMV infection, since they employ a different mechanism of action. Our cell viability assays demonstrate that the effective peptides have no statistically significant toxicity at the highest concentrations tested in our studies (data not shown). Consequently, we do not expect adverse effects or toxicity due to treatments developed from these synthetic peptides.

## Methods

### Design and Synthesis of peptides

WWIHS is an experimentally determined algorithm that can be used to estimate the propensity of amino acid sequences to interact with lipid membrane interfaces [49]. Sequences of HCMV gB with positive WWIHS score were identified using Membrane Protein explorer (MpeX version 3.0) (http://blanco.biomol.uci.edu/mpex), a computer program based on WWIHS. HCMV gB synthetic peptides were synthesized by solid phase conventional N-α-9-fluorenylmethyloxycarbonyl chemistry by Genemed Synthesis Inc. (San Francisco, CA). Peptides were purified by reverse-phase high performance liquid chromatography and confirmed by amino acid analysis and electrospray mass spectrometry. Peptide stock solutions were prepared in 10% dimethyl sulfoxide (DMSO, spectroscopy grade): 90% (v/v) H_2_O. Peptide concentrations were determined by absorbance of aromatic side chains at 280 nm (SmartSpec ™ 3000, BioRad, Hercules, CA).

### Viruses and cells

The Towne strain of HCMV containing the green fluorescent protein (GFP) expression cassette was obtained from Dr. Daniel Streblow at the Oregon Health Science University and was propagated in human foreskin fibroblasts (HFF). Viral supernatants were collected 5 days after 100% CPE was observed, centrifuged to clear cell debris, and filtered through a 0.45 μm filter. HFF were grown in Dulbecco's modified Eagle medium (DMEM) supplemented with 10% fetal bovine serum (FBS), penicillin G (100 U/mL), streptomycin (100 mg/mL), and GlutaMAX (2 mM).

### Viral infectivity assays

HFF were seeded at a density of 3.5×10^5 ^cells in each well of a 24-well plate 24 hours prior to infection. HFF were washed with 1× DPBS and mock- or virus-infected for 90 minutes at RT with the Towne-GFP strain of HCMV (0.5 MOI) preincubated with different concentrations of inhibitory peptides at 37°C for 90 minutes. After infection, virus was removed and Dulbecco's modified Eagle medium (DMEM) supplemented with 10% fetal bovine serum (FBS), penicillin G (100 U/mL), streptomycin (100 mg/mL), and GlutaMAX (2 mM) was added to each well and cells were incubated at 37°C for 48 hours. GFP-positive cells were visualized 48 hours post-infection by fluorescence microscopy and then quantified using flow cytometry.

### Flow Cytometry

HFF cells were trypsinized, centrifuged, and resuspended in 1% FBS DPBS. GFP-positive cells were quantified using flow cytometry (Cytomics FC 500 Beckman Coulter, Fullerton, CA).

## List of abbreviations

HCMV: Human cytomegalovirus; gB: glycoprotein B; HFF: human foreskin fibroblasts; MOI: multiplicity of infection; WWIHS: Wimley-White Interfacial Hydrophobicity Scale

## Competing interests

The authors declare that they have no competing interests.

## Authors' contributions

LM participated in the experimental design, performed viral propagation and all experiments, and drafted the manuscript. CM and RG conceived of the study, and participated in its design and assisted LM in the interpretation of results. All authors read and approved the final manuscript.
